# AI Approaches towards Prechtl’s Assessment of General Movements: A Systematic Literature Review

**DOI:** 10.3390/s20185321

**Published:** 2020-09-17

**Authors:** Muhammad Tausif Irshad, Muhammad Adeel Nisar, Philip Gouverneur, Marion Rapp, Marcin Grzegorzek

**Affiliations:** 1 Institute of Medical Informatics, University of Lübeck, Ratzeburger Allee 160, 23562 Lübeck, Germany; muhammad.nisar@student.uni-luebeck.de (M.A.N.); gouverneur@imi.uni-luebeck.de (P.G.); grzegorzek@imi.uni-luebeck.de (M.G.); 2 Punjab University College of Information Technology, University of the Punjab, Lahore 54000, Pakistan; 3 Clinic for Pediatric and Adolescent Medicine, University of Lübeck, Ratzeburger Allee 160, 23562 Lübeck, Germany; marion.rapp@uni-luebeck.de

**Keywords:** general movement assessment, fidgety movements, cerebral palsy, motion sensors, visual sensors, multimodal sensing, physical activity assessment, machine learning, artificial neural network

## Abstract

General movements (GMs) are spontaneous movements of infants up to five months post-term involving the whole body varying in sequence, speed, and amplitude. The assessment of GMs has shown its importance for identifying infants at risk for neuromotor deficits, especially for the detection of cerebral palsy. As the assessment is based on videos of the infant that are rated by trained professionals, the method is time-consuming and expensive. Therefore, approaches based on Artificial Intelligence have gained significantly increased attention in the last years. In this article, we systematically analyze and discuss the main design features of all existing technological approaches seeking to transfer the Prechtl’s assessment of general movements from an individual visual perception to computer-based analysis. After identifying their shared shortcomings, we explain the methodological reasons for their limited practical performance and classification rates. As a conclusion of our literature study, we conceptually propose a methodological solution to the defined problem based on the groundbreaking innovation in the area of Deep Learning.

## 1. Introduction

Movements of the human body look very simple but consist of complex coordination systems, subsystems, and monitoring pathways. Any disorder in the coordination system like progressive neuromuscular disorders, injuries to the brain, and genetic disorders can create problems in movement and posture. For example, cerebral palsy (CP) describes a group of disorders of lifelong physical disability caused by a non-progressive brain injury or lesion acquired during the antenatal, perinatal, or early postnatal period [1]. The severity, patterns of motor involvement, and associated impairments, such as communication, intellectual ability, and epilepsy, vary widely and persist across the life course [2]. In addition, neonatal mortality has decreased in preterm infants in the past decade, extremely preterm infants (born at <27 gestational weeks) remain at the highest risk for neonatal morbidity and the occurrence of CP [3]. Therefore, the prevalence of CP has remained stable over the last forty years at 2–3 per 1000 live births in countries with a developed health care system.

At present, there are no uniform clinical procedures for the prediction of motor impairments like CP in high-risk infants and the recognition of those at the highest risk generally requires the combination of clinical history, various clinical assessments and expertise of the observer [4]. Some studies, e.g., [5,6,7], have exposed the fact that early recognition of motor impairment leads to early interventions that might reduce the severity of the motor impairment and the restrictions in daily activities [8].

Prechtl presented the General Movements Assessment (GMA) as a valuable tool for the prediction of cerebral palsy in high-risk infants [9,10]. General movements (GMs) are spontaneous movements of infants up to five months post-term involving the whole body. The movements vary in sequence, speed, and amplitude. Depending on the infant’s age, one distinguishes between the general movements (GMs) (preterm general movements (∼28–36/38 gestational weeks) or term/writhing movements (36/38–46/52 gestational weeks)), and the fidgety movements (FMs) (46/50–55/60 gestational weeks) [9]. Next to normal GMs and normal FMs (F+ or F++), one distinguishes between poor repertoire GMs (PR) with a monotonous sequence of movements and reduced variance in speed and amplitude of movements, cramped synchronized GMs (CS) which appear stiff with bilateral contraction and relaxation of the legs and the abdominal wall, and chaotic GMs (Ch) which appear jerky, rowing, fast, and have a large amplitude. The non-normal FMs comprise abnormal FMs (AF) with large amplitude, fast and jerky movements, as well as the absence of FMs (F−). Showing cramped synchronized or chaotic GMs around term or the absence of fidgety movements (F−) at 3 to 5 months post-term have an excellent predictive value for cerebral palsy [11,12]. However, the assessment is based on videos of the infant that are rated by trained professionals, therefore, the method is time-consuming and expensive.

As a result of the nominal use of GMA in neonatal follow-up programs, several studies have tried to automate this method. These studies are based on either indirect sensing using visual sensors (2D or 3D video) [7,13,14,15,16,17,18,19,20,21,22,23,24,24], direct sensing using motion sensors [25,26,27,28,29,30,31], or both [32,33,34]. They have shown excellent results, however, they lack full automation and also have several fundamental limitations. First, all the studies are either based on a small number of subjects or a fewer number of data samples with respect to CP [7,18,19,20,25,26,27,32,34]. It is also not clear if the prediction model in these studies has external validity for high-risk infants. Second, the research work in some studies is based on convenience samples that do not reflect the usual clinical cohorts. Third, the movement features used in previous studies lack generality due to less number of subjects and examples. Lastly, all the reviews, except [17,20,23,24,35,36], are not using state-of-the-art Deep Learning (DL) algorithms to automate the GMA process. The DL algorithms are popular approaches of Artificial Intelligence (AI) which not only provide a generalized solution but also perform well for accurate detection of the classes in visual and time-series data. Therefore, an end-to-end system is needed to analyze the infant’s movements in the early infancy.

There are some related review articles for monitoring body movements of infants using sensor technology. Chen et al. [37] outlines the wearable sensor systems for monitoring body movements of neonates apart from visual sensors and state-of-the-art AI algorithms for the development of an automated end-to-end system. Zhu et al. [38] present a broad overview of wearable sensors intending to measure various types of physiological signals of infants. The authors in [39] discuss state-of-the-art movement recognition technology for assessing spontaneous general movements in high-risk infants, however, they do not focus on the design and development of the system. They discuss the wearable and visual sensors averagely. Zhang [40] review machine learning methods in cerebral palsy research and evaluates algorithms in movement assessment for CP prediction.

The primary objective of this article is to systematically analyze and discuss the main design features of all existing technological approaches trying to classify the general movements of infants and explain the methodological reasons for their limited practical performance and classification rates. The main contributions of this paper can be summarized as follows:

- We present a structured review of the current technological approaches that detect general movements and/or fidgety movements, and categorize them according to the AI techniques they use. We slice up these approaches into three vital categories: visual sensor-based, motion sensor-based, and multimodal (fusion of visual and motion sensory data).
- We categorize and present a summary of the sensor technology and classification algorithms used in the existing GMA approaches.
- We also present a comparative analysis of reviewed AI-based GMA approaches with respect to input-sample size, type of features, and classification rate.

Prior to continue, it is worth noting that the correct classification of GMs is a difficult task and relies on clinical expertise. While some previous (machine learning) studies evaluated the ground truth of their data by introducing trained GMA experts, some recognized ambiguous, arbitrary, or incorrect classification or did not present detailed information about the realized process. In order to provide an objective overview, we nevertheless indicate the classes and terms specified in the papers and highlight if the classification was not carried out properly. Moreover, this article does not talk about preprocessing operations, for example (image enhancement, noise attenuation, finding the region of interest, etc.), since they fall outside from the scope of this article. In addition, we duly note that understanding this paper requires knowledge of machine learning concepts and performance evaluation techniques of classifiers. An extensive but straightforward explanation of these concepts can be found in [41,42].

This article is organized as follows: Section 2 describes the review methodology. Section 3 lists and describes the sensor modalities applied for GMA. Section 4 lists and outlines the classification algorithms used in the reviewed GMA. Section 5 details the GMA based on the visual sensors, motion sensors and multimodal sensors. Finally, Section 6 concludes this paper and provides ideas for future research activities in this area.

## 2. Methods

### 2.1. Literature Search Strategy

The primary aim of this paper was to provide a review on the main design features of the existing technological approaches dealing with the classification of the general movements of infants. The paper also explains the methodological reasons for their limited practical performance and classification rates. The potential research articles were searched on PubMed, IEEE Xplore, Microsoft Academic, and Semantic Scholar. As a result of the discrete search patterns of aforementioned databases and search engines, we used slightly different strings for each of search queries. Our search strategy for PubMed database is shown in Table 1.

### 2.2. Literature Selection Strategy

Our selection strategy was implemented in two phases. In the first phase, all the authors read abstracts of the papers and excluded all that deal with the neurological problems of the infants other than the early detection of cerebral palsy.

In the second phase, authors read the full text of the papers and performed selection by implementing the following inclusion criteria.

- Whether the paper presented a study of infants.
- The infants should be in the age group relevant to general and fidgety movements.
- The studies should have used video and/or motion sensors.
- The studies should have implemented machine learning (or statistical) approaches.

### 2.3. Screening Strategy

**Figure 1:**
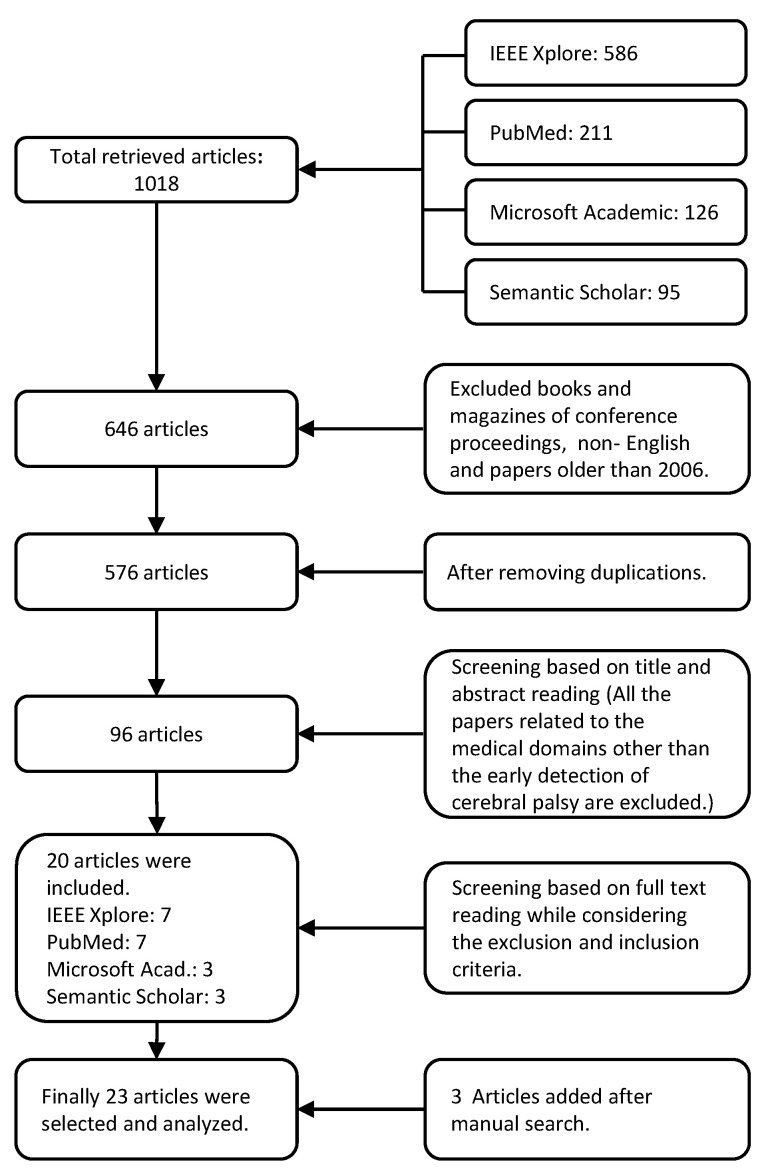
The procedure of literature selection and screening.

We found 1018 potential research articles. We excluded books and magazines of conference proceedings, non-English articles, and the papers not falling within the time period of 2006–2020. After the removal of duplication, we selected 576 articles. Figure 1 shows the complete screening process. We performed our first phase of selection on 576 articles by reading their titles and abstracts, and excluded all that do not deal with the early detection of cerebral palsy. Therefore, the article count is reduced to 96. We read the full text of these 96 articles and finally selected 20 articles based on the inclusion criteria as mentioned in Section 2.2. Three articles were included after manual searching. Finally 23 articles were considered in this review. All the authors took part in the screening strategy.

## 3. Sensor Modalities Used for General Movement Assessment

The advancement in sensor technology facilitates the automatic monitoring of infants’ movements. Hence, a system using visual or motion sensors can be useful to track these movements to diagnose motor impairments at early stages. This section briefly describes the sensor modalities used in the reviewed studies. Table 2 specifies the sensor modalities used by a particular GMA study.

**RGB Camera** records the color information at the time of exposure by evaluating the spectrum of colors into three channels, i.e., red, green, and blue. They are easily available, portable, and suitable for continuous assessment of infants in clinics or at home due to their contact-less nature comparing with other modalities. Various motion estimation methods for example, Optical Flow, Motion Image, can be used for RGB videos.
**Vicon System** is an optoelectronic motion capture system based on several high-resolution cameras and reflective markers. These markers are attached to specific, well-defined points of the body. As a result of body movement, infrared light reflects into the camera lens and hits a light-sensitive lamina forming a video signal. It collects visual and depth information of the scene [43].
**Microsoft Kinect** sensor consists of several state-of-the-art sensing hardware such as RGB camera, depth sensor (RGB-D), and microphone array that helps to collect the audio and video data for 3D motion capture, facial, and voice recognition. It has been popularly used in research fields related to object tracking and recognition, human activity recognition (HAR), gesture recognition, speech recognition, and body skeleton detection. [44].
**Accelerometers** are sensing devices that can evaluate the acceleration of moving objects and reveal the frequency and intensity of human movements. They have been commonly used to monitor movement disorders, detect falls, and classify activities like sitting, walking, standing, and lying in HAR studies. Due to small size and low-price, they have been commonly fashioned in wearable technologies for continuous and long-term monitoring [45,46].
**Inertial Measurement Unit (IMU)** is a sensory device that provides the direct measurement of multi-axis accelerometers, gyroscopes, and sometimes other sensors for human motion tracking and analysis. They can also be integrated in wearable devices for long term monitoring of daily activities which can be helpful to assess the physical health of a person [47].
**Electromagnetic Tracking System (EMTS)** provides the position and orientation quantities of the miniaturized sensors for instantaneous tracking of probes, scopes, and instruments. Sensors entirely track the inside and outside of the body without any obstruction. It is mostly used in image-guided procedures, navigation, and instrument localization [48,49].

## 4. Classification Algorithms Applied for General Movement Assessment

**Figure 2:**
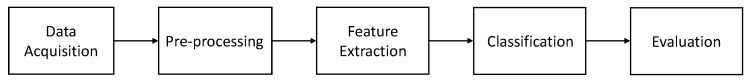
This figure shows necessary steps to solve a classification problem.

In machine learning, classification and regression algorithms are used to predict results based upon input data. A classification categorizes the data into predefined classes, whereas regression estimates an outcome from a set of input data. These algorithms are implemented in two phases—training and testing. In each of these phases, the raw data are acquired by sensors. After pre-processing the data, suitable features are extracted to build feature vectors. The feature vectors can be split into train and test datasets. In the training phase, the train dataset is used to train a model. In the testing phase, the trained model is used to predict the results of feature vectors belonging to the test dataset. Finally, the performance of the model is evaluated using different matrices on the test data. Figure 2 shows the essential stages of classification.

Sensors used in data acquisition process for the assessment of GM and FM studies are shown in Table 2. Features extraction process is out of the scope of our topic. However, the classification algorithms used by a particular study are shown in Table 3. The outcomes of classification procedure in the reviewed studies are shown in Table 4, Table 5 and Table 6.

In general, a classification algorithm evaluates the input features to make a decision or diagnosis. The selection of the algorithm depends on many factors, for example, type of data, size of data, and available resources to process the data. This section provides the description of classification algorithms used in GMA studies for the discrimination of infant’s movements or impairments.

**Naive Bayes (NB)** belongs to the group of probabilistic classifiers based on implementing the Bayes’ theorem with the simple assumption of conditional independence that the value of a feature is independent of the value of any other feature, and each feature contributes independently to the probability of a class. NB combines the independent feature model to predict a class with a common decision rule known as maximum likelihood estimation or MLE rule. Despite their simplicity, NB classifiers performed well on many real-world datasets such as spam filtering, document classification, and medical diagnosis. They are simple to implement, need a small amount to training data, can be very fast in prediction as compared to most well-known methods [50].
**Linear Discriminant Analysis (LDA)** is used to identify a linear combination of features that splits two or more classes. The subsequent combination can be used as a linear classifier or dimensionality reduction step before the classification phase. LDA is correlated to principal component analysis (PCA), which also attempts to find a linear combination of best features [51]. However, PCA reduces the dimensions by focusing on the variation in data and cannot form any difference in classes. In contrast, it maximizes the between-class variance to the within-class variance to form maximum separable classes [52].
**Quadratic Discriminant Analysis (QDA)** is a supervised learning algorithm which assumes that each class has a Gaussian distribution. It helps to perform non-linear discriminant analysis and believes that each class has a separate covariance matrix. Moreover, It has some similarities with LDA, but it cannot be used as a dimensionality reduction technique [53].
**Logistic Regression (LR)** explores the correlation among the independent features and a categorical dependent class labels to find the likelihood of an event by fitting data to the logistic curve. A multinomial logistic regression can be used if the class labels consist of more than two classes. It works differently from the linear regression, which fits the line with the least square, and output continuous value instead of a class label [54].
**Support Vector Machine (SVM)** is a supervised learning algorithm that analyzes the data for both classification and regression problems. It creates a hyperplane in high dimensional feature space to precisely separate the training data with maximum margin, which gives confidence that new data could be classified more accurately. In addition to linear classification, SVM can also perform non-linear classification using kernels [55].
**K-Nearest Neighbor (KNN)** stores all the training data to classify the test data based on similarity measures. The value of K in the KNN denotes the numbers of the nearest neighbors that can involve in the majority voting process. Choosing the best value of k is called parameter tuning and is vital for better accuracy. Sometimes it is called a lazy learner because it does not learn a discriminative function from the training set. KNN can perform well if the data are noise-free, small in size, and labeled [56].
**Decision Tree (DT)** is a simple presentation of a classification process that can be used to determine the class of a given feature vector. Every node of DT is either a decision node or leaf node. A decision node may have two or more branches, while the leaf node represents a classification or decision. In DTs, the prediction starts from the root node by comparing the attribute values and following the branch based on the comparison. The final result of DT is a leaf node that represents the classification of feature vector [57].
**Random Forest (RF)** is an ensemble learning technique that consists of a collection of DTs. Each DT in RF learns from a random sample of training feature vectors (examples) and uses a subset of features when deciding to split a node. The generalization error in RF is highly dependent on the number of trees and the correlation between them. It converges to a limit as the number of trees becomes large [58]. To get more accurate results, DTs vote for the most popular class.
**AdaBoost (AB)** builds a robust classifier to boost the performance by combining several weak classifiers, such as a Decision Tree, with the unweighted feature vectors (training examples) that produce the class labels. In case of any misclassification, it raises the weight of that training data. In sequence, the next classifier is built with different weights and misclassified training data get their weights boosted, and this process is repeated. The predictions from all classifiers are combined (by way of majority vote) to make a final prediction [59].
**LogitBoost (LB)** is an ensemble learning algorithm that is extended from AB to deal with its limitations, for example, sensitivity to noise and outliers [60]. It is based on the binomial log-likelihood that modifies the loss function in a linear way. In comparison, AB uses the exponential loss that modifies the loss function exponentially.
**XGBoost (XGB)** or eXtreme Gradient Boosting is an efficient and scalable use of gradient boosting technique proposed by Friedman et al. [60], available as an open-source library. Its success has been widely acknowledged in various machine learning competitions hosted by Kaggle. XGB is highly scalable as compared with ensemble learning techniques such as AB and LB, which is due to several vital algorithmic optimizations. It includes a state-of-the-art tree learning algorithm for managing sparse data, a weighted quantile method to manage instance weights in approximate tree learning—parallel and distributed computing for fast model exploration [61].
**Log-Linearized Gaussian Mixture Network (LLGMN)** is a feed-forward kind of neural network that can estimate a posteriori probability for the classifications. The network contains three layers and the output of the last layer is considered as a posteriori probability of each class. The Log-Linearized Gaussian Mixture formation is integrated in the neural network by learning the weight coefficient allowing the evaluation of the probabilistic distribution of given dataset [62].
**Convolutional Neural Network (CNN)** is a class of ANN, most frequently used to analyze visual imagery. It consists of a sequence of convolution and pooling layers followed by a fully connected neural network. The convolutional layer convolves the input map with *k* kernels to provide the *k*-feature map, followed by a nonlinear activation to *k*-feature map and pooling. The learned features are the input of a fully connected neural network to perform the classification tasks [63].
**Partial Least Square Regression (PLSR)** is a statistical method that uncovers the relationship among two matrices by revealing their co-variance as minimum as feasible, Rahmati et al. [33] apply it to predict cerebral palsy in young infants. Here, PLSR uses a small sequence of orthogonal Partial Least Square (PLS) components, specified as a set of weighted averages of the X-variables, where the weights are evaluated to maximize the co-variance with the Y-variables and Y is predicted from X via its PLS components or equivalently [33,64].
**Discriminative Pattern Discovery (DPD)** is a specialized case of *Generalized Multiple Instance* (GMI) learning, where learner uses a collection of labeled bags containing multiple instances, rather than labeled instances. Its main feature is to solve the weak labeling problem in the GMA study by counting the increment of each instance in order to classify it into three pre-defined classes. Moreover, DPD performs the classification based on the softs core proportion rather than a hard presence/absence criteria as in conventional GMI approaches [28].

## 5. Methodology of the Reviewed Approaches

**Figure 3:**
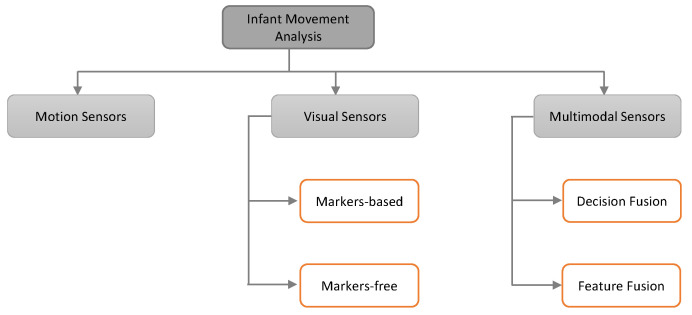
This figure shows the tree diagram of the infant’s General Movements Assessment (GMA) methods based on three different categories of sensors. It further categorizes visual sensors-based methods into marker-based and marker-free. It also divides multimodal sensors-based methods into decision and feature fusions.

The automated analysis of GMs and FMs of infants is an emerging topic in Artificial Intelligence because of their rising demand for objective assessment in the clinical environment and homecare. Various methods are available to automatically identify infants GMs and FMs relying on visual sensors, motion sensors, or multimodal sensors. We further categorize these methods as shown in Figure 3.

### 5.1. General Movement Assessment Based on Motion Sensors

Motion and wearable sensors have been popularly used to detect physical activities in health care systems [65,66]. Motion sensors, for example, accelerometers, gyroscopes, and magnetometers provide satisfactory data quality and reliability for the assessment of movement disorders. Moreover, they are affordable, necessarily miniaturized, and improve more rapidly compared to alternative devices usually used for movement assessment [67,68]. Their use ranges from observing functional motor movements, i.e., neuromuscular disorders (stroke and Parkinson’s disease) to the evaluation of physical activities to identify disease patterns for prevention, therapy, rehabilitation, and additionally, the assessment of changes in the movement of the newborn [26,27,67,68].

In recent times, wearable sensor technology has been used for capturing and analyzing spontaneous GMs and FMs of infants without the need of a clinical observer. Singh and Patterson [25] proposed a system that consists of accelerometers to analyze abnormal movements of infants. The data was acquired from ten premature babies. They showed very good classification results. However, their study has several limitations. For example, the sample size is too small having only premature babies. The study needs manual annotation by trained specialists using video recordings. Furthermore, they classify the normal vs. abnormal movements based only on the presence and absence of CS. They did not provide any clinical evidence to show the effectiveness of their study. Gravem et al. [27] proposed a system to monitor GMs in preterm infants using five accelerometers that were embedded in cloth bands and placed around the limbs and forehead. The infants were also filmed at the same time. The collected data were annotated manually based on visual observation. After data preprocessing, they computed 166 statistical and temporal features. Their proposed model was able to identify CS with 70–90% accuracy. To validate their claims, further study with more data and clinical follow-up outcome is necessary. Authors in [30] presented a model to recognize the CS of 10 preterm infants from accelerometers data. They extracted several statistical features such as mean, max, min, standard deviation, and temporal features for each measurement. The analysis was conducted by applying Area-Under-the-Curve (AUC), which showed that AdaBoost applied to Naive Bayes classifiers is a distinctly accurate classifier. Furthermore, they also compared the Erlang-Cox Dynamic Bayes Networks models and claimed that they are equal in terms of AUC. Heinze et al. [26] extracted 32 features based on velocity and acceleration derived from the accelerometer data. Their dataset was recorded from 23 infants (19 healthy and 4 high-risk). They use Decision Tree classifier and obtain an overall accuracy of 89%. However, their dataset seems to be unbalanced because the number of high-risk infants in this study was substantially less than the number of healthy infants. Moreover, they used wired accelerometers to collect the spontaneous movement of infants which might cause hindrance to the free movements.

Karch et al. [69] provided the first study to capture the infant’s limbs movements using the Electromagnetic Tracking System (EMTS) to calculate the segmental kinematics. They recorded the data of 20 infants between term and post-term age by attaching four sensors to the right arm and four sensors to the right leg of the body. Their proposed body model consists of three segments for each limb, and they represented each segment by the Cartesian coordinate system. After calculating the joint centers and the position of the rotation for each joint center from the captured movement data, they use the root mean square deviation (RMSD) of the total Least Square Regression (LSR) to measure the calibration movements of infant’s limbs at calibration time. In addition to the small sample size, this study shows the potential to use EMTS for infant’s movements analysis; further studies are necessary to investigate the distinctive features from the recorded data that can help in the decision support. In order to provide an objective analysis, it is necessary to quantify the movement features. Karch et al. [70] recorded the movements of 53 preterm and term infants using the EMTS to compute a complexity score from all segments of movements. Their automated approach detected the complex segments marked by the physicians with an accuracy of 77%.

Interestingly, Philippi et al. [29,31] computed 3 kinematic features from them with repetitive movement in the upper limbs identified as finest predictor of CP. However, the accelerometer and magnetic tracking system used by [26,29,31] were wired and massive, causing significant practical problems. Authors in [32] collected two sets of data from 78 infants at 10–18 weeks post-term using miniBird motion sensors and a video camera. After the motion segmentation, they extracted the following three features: area out of standard deviation (STD) from moving average, periodicity, and correlation between trajectories. They achieved 85% accuracy on sensor data with SVM classifier. Despite the good accuracy, however the only limitation, user at first need to label some motion trajectories. Rahmati et al. [33] claim that when there are relatively few subjects but several viable features, the machine learning algorithm may lead to a suboptimal solution. Therefore, they performed frequency-based analysis of data acquired by accelerometers attached to the limbs of the infants. The data from 78 infants was examined to select suitable set of features. A cross-validation technique with Partial Least Square Regression (PLSR) was applied to estimate the predictability of the model. Furthermore, they also claim that the frequency between 25–35 Hz was found to be most meaningful.

In this section, we discussed the infant’s GMA using motion sensors that includes Electromagnetic Tracking System and wearable sensors (accelerometers and IMUs). These sensors pose a high temporal resolution and high availability. In addition, they are low cost and privacy-preserving in case of wearable sensors. Therefore, they can be used for comprehensive analysis. In contrast, the assessment under Electromagnetic Tracking System is expensive, requires complex setup, and is not suitable for clinics and homecare.

### 5.2. General Movement Assessment Based on Visual Sensors

Over the past few years, motion analysis has acquired numerous attention due to the technological evolution and exponential demand for robust, more advanced systems to capture the human body movements for clinical or behavior assessment and other applications [71,72,73,74,75]. Visual-based systems either rely on markers attached to certain body parts or explore marker-free solutions by using image features, for example (color, shape, edges, etc.) to encode the motion information from video data. While both approaches have their own benefits, both have certain limitations as well. A comparison of marker-based and marker-less motion capture for gait analysis [76] and ergonomics [77] already exist. While marker-based techniques have been proven in literature to be relatively accurate, specific markers and hardware are needed to detect a reasonable number of markers simultaneously. In contrast, marker-free approaches give up some of the accuracy in exchange for the freedom of using no markers or a specific setup on the tracked individual [78].

In the following, the two different methods are explained in more detail and evaluated with regard to their application for GMA.

#### 5.2.1. Marker-Based Approaches

Marker-based motion capture is a prevalent method used for human movement analysis in which spatio-temporal variations in the point of markers attached to the body allow to quantitatively describe body motion in the computer. Often markers are placed and tracked at joints’ location to reconstruct the body pose. To recognize markers, several image-based techniques exist. Passive markers can be located via color [79] or a combination of infrared stroboscopic illumination and retro-reflective markers [80]. Little light sources, like light emitting diodes (LEDs), have been used as active markers before [81]. The main concern using these techniques is to track a sufficient number of markers for the pose reconstruction, which can be easy covered or overlapped.

One of the first studies to detect CP using a marker-based system was carried out by Meinecke et al. [7]. The authors proposed an analysis system for infants by using 20 reflective markers and 7 infrared cameras to capture 3D motion. Five experienced physicians and additional literature [82,83,84,85] were consulted to gather key parameters for the analysis of spontaneous motor activity of 22 infants. Further statistical and mathematical parameters were computed to yield a total of 53 quantitative features. Using cluster analysis with Euclidean distance, a combination of the eight most significant features to distinguish healthy and affected infants were found. This optimal feature space was then used as an input to the quadratic discriminant analysis algorithm to acquire an overall detection rate of the classification methodology (73% accuracy to detect healthy and affected participants). However, such type of 3D motion capture systems are costly, challenging to set up, not easily portable, and have high computational complexity which limits their clinical applicability. The work of Berthouze and Mayston [86] focused on establishing surface-cluster to access general movement especially focusing on the quantification of joint rotations. Self-cut polycarbonate sheet frames to cluster markers (also referred to as cluster) were evaluated during a validation study with soft-body dummy dolls and a case study consisting of 4 typically developing infants. To overcome the problem of very young infants having insufficient space at their shanks, each frame comprises 3 or 4 markers. No disruptive overlapping or covering of markers could be accounted using a 6-camera setup. Despite the low number of infants during the case study, several general conclusions could be phrased. Robust estimation of joints, especially angular motion of hip and knee rotations, could be extracted by using the proposed cluster. The setup time compared to using simple markers and the risk of removing markers through movement was reduced. The authors “suggest that this surface-marker cluster approach makes it possible to fully quantify infants’ general movements” [86]. Nevertheless, the use of multiple cameras seems to be expensive and more complex in terms of preparation time compared to approaches using one RGB video. The authors in [87] also used reflective markers, but only a digital camera to capture the infant’s movements. They computed and tested different kinematic features, such as the cross-correlation of velocities and accelerations between limbs from 2D videos. They also found that the movements of infants who later develop CP were jerkier than those of healthy ones. The drawback of this method is the estimation of velocity in pixels/frame. As the distance between camera and infant is unidentified, it is not possible to convert the dimensions from pixels to other units of measurement. Furthermore, they also overlook the movement perpendicular to the camera which makes their approach invariant to scaling. These issues can be addressed by using a depth camera.

In a most recent survey, Colyer et al. [88] provided the evaluation of several marker-based systems. They are complex to install and fine-tune. Although the author in [88] was not focused on infants, we can assume due to the sensitivity and size of infants that increasing the markers on the infant’s body parts can increase the complexity when markers are close to each other. Furthermore, the case of increasing IR-based markers would make the system more expensive.

#### 5.2.2. Marker-Free Approaches

Over the last decade, marker-free approaches have become very attractive in the research community for various applications of computer vision. Instead of applying specific markers on the human body, they make use of image features like shape, edges, and pixel location to detect and track the human body parts. Marker-free techniques have the advantage that they do not intervene and therefore do not interfere with the spontaneous movements of the infants. Often stationary digital video cameras are placed above the infant to record it in supine position, being awake but not distracted. Cameras can be distinguished in mainly two section, simple RGB and depth cameras. Furthermore, deep learning approaches have been summarized in an additional paragraph.

**RGB Cameras:** Adde et al. [22] were the first to use computer-based video analysis to classify infants’ movements according to the GMA. A total of 137 video recordings of 82 infants (10–18 weeks post-term age) were labeled with observable FMs or not observable FMs according to the GMA (119 with and 27 without FMs). A General Movement Toolbox (GMT) was implemented to view, crop, preprocess, and extract features to classify videos into non-fidgety or fidgety. The analysis was mainly built upon calculated motion images, where each pixel represent whether there is movement or not. From this several features, for example the quantity of motion as “the sum of all pixels that change between frames in the motion image divided by the total number of pixels in the image” [22], were extracted. It could be shown that the videos of infants lacking FMs had a significant lower mean quantity of motion compared to infants with FMs. Furthermore, the variability of centroid was determined to have the strongest association with the absence of FMs across all tested variables using a logistic regression. In conclusion, it has been shown that a non-intrusive computerized analysis can yield features associated with the absence of FMs. Thus, showing that the GMA could be replaced in theory. In [14], the authors further extended their work to predict CP as well. They used 2D videos and a simple frame differencing software without any instrumentation to calculate a motion image. Several hand-crafted features from motion images in addition to the velocity and acceleration of the centroid of the motion were extracted. The best performance was achieved using a cerebral palsy predictor (CPP), consisting of a combination of the centroid of motion standard deviation, the quantity of motion mean, and the quantity of motion standard deviation computed from the motion image. CP was predicted with 85% sensitivity and 88% specificity. The development outcome was defined as an examination at 4–7 years of age. While the performance metrics look promising, the small sample size for this study of 30 high-risk infants can be questioned. Using recordings of 150 infants (10–15 weeks), Støen et al. [89] elevated their work by incorporating sporadic FMs as well. These recently defined movements characterized by short FMs (1–3 s) with up to 1-min intermediate pauses and the absence of FMs were accounted for 48 of the infants by two certified observers. The absence of normal FMs could be associated with a large variability of the spatial center of movements. In contrast, normal FMs lead to an evenly distributed movement and thus for a more stable center of motion. Additionally, they showed that it is not possible to distinguish between healthy and abnormal movement based on the quantity of motion, as it is not correlating with the presence of FMs. Further automated analysis of sporadic FMs could help to understand their nature, as it is not clear whether they are clinically relevant or not [90]. Stahl et al. [16] also recorded 2D videos but an optical flow method to detect moving objects within the scene was realized. The optical flow provides the speed and direction of the object as compared with a frame-differencing method. Visible differences between healthy and affected children were recognized by plotting only the *x* or *y* components of the movement trajectories. While healthy children had smaller and more frequencies in their components, there were parts of no movement and a more discontinuous signal over time for affected infants. Moreover, they computed wavelet and frequency features and identified three feature values for the analysis of FMs using a support vector machine. By using a 10-fold cross-validation, they achieved 93% accuracy to distinguish impaired from unimpaired infants. In this study, the use of 3 features is questionable, and study samples in terms of the number of children with CP (15 infants with and 67 infants without CP) are too small. Furthermore, the proposed data analysis methods [14,16] are sensitive to lighting conditions, cloths, and skin color. Dai et al. [21] evaluated the use of a Kernel Correlation Filter (KCF) [91] to track trajectories of the limbs and whole body of infants to classify their movement as normal or abnormal. Motion trajectories were split in their X and Y components and the X axis discarded for later computations. Features were extracted using Discrete Wavelet Transform (DWT) which considers both frequency and time information and calculation of the square of the amplitude spectrum to retrieve a characteristic of the energy of the signal (power spectrum). PCA was then applied to reduce the dimensionality of the features space. The authors were the only ones to implement Stacking, a type of Ensemble Learning where classifiers are piled in layers. They created a stacked training model consisting of SVM, RF, and AB in the first layer, feeding their output to a second layer consisting of XGBoost which yields the final classification. In addition, a model for the wavelet and power spectrum features each and a weighted combination has been trained. Testing on 120 video samples (60 normal-behavior, 60 abnormal infants, age 10–20 weeks) a best accuracy of 93.3% with the combined model was achieved. Although it could be shown that KCF and Stacking yield high accuracy in classifying normal and abnormal behavior, no detailed information about the involved ground truth is given.

In clinical observations, CS and FM are early markers for later development of CP [11,92]. Therefore, to get a good feature set that represents the full clinical insight, the authors in [32] implemented a motion segmentation method proposed in [93]. They collected a dataset of 78 infants recorded with a 2D monocular camera. They also captured motion sensor data simultaneously. The authors computed the dense trajectories by using the Large Displacement Optical Flow (LDOF) and then applied a graph-based segmentation algorithm to segment them into groups of individual body parts. Three types of features (area out of standard deviation (STD) from moving-average, periodicity, and correlation between trajectories) proposed in [7] were extracted. The first two features were chosen to detect a lack of fluent movement, the last one to detect high correlation between two limbs which can be a predictor for CP [92] and abnormal behavior [94] respectively. By using a Support Vector Machine (SVM), they got 87% accuracy on the motion segmentation dataset. Despite the excellent accuracy, the user must label a small number of trajectories. Rahmati et al. [33] made use of the same dataset, as mentioned in [32], to propose an intelligent feature set for the prediction of CP. They extracted the motion data out of video by using the similar method proposed in [93]. A Fast Fourier Transform (FFT) to extract the final feature from motion data was applied. The authors computed 2376 features from video data and performed a Partial Least Square Regression (PLSR) along with a cross-validation to estimate the predictability of the model. They claim that they achieved 91% accuracy for their CP prediction. These results should be received with caution, as the number of children with CP (14 infants) is very low compared to the one without CP (64 infants). Such a class imbalance can introduce certain tendencies towards the dominant class in classifier and the evaluation by accuracy can be misleading [32,33]. Orlandi et al. [13] screened 523 videos of babies at 3–5 months corrected age and selected 127 of them for automatic analysis. During the selection process several criteria, for example if the complete infant is always visible or light conditions, were checked. Each infant was categorized by a certified observer according to the criteria described by Hadders-Algra [95] having typical (98 infants) or atypical (29 infants) movements. The creation of the automated system included 5 steps: a motion estimation with LDOF which uses pixel displacement between frames, an infant segmentation to remove the background, feature extraction of 643, feature selection to reduce the number of features, and classification. Using a Leave-one-out cross-validation (LOO-CV) several classifiers (Logistic, AdaBoost, LogitBoost, and Random Forest) were trained to distinguish between “typical” vs. “atypical” movement and “CP” vs. “no CP”. While the best accuracy of 85.83% for the GMA was achieved with the AdaBoost classifier, the Random Forest yielded the best result (92.13% accuracy) in classifying CP even outperforming the clinical GMA itself (85.04% accuracy). Being one of the first studies to include more than 100 preterm infants in their tests, Orlandi et al. [13] show that an automated procedure could possibly replace the clinical GMA. Moreover, Random Forest and AdaBoost seem to be a good choice of classifier, but the method lacks kinematic features that could be introduced by using depth cameras. A new model called Computer-based Infant Movement Assessment (CIMA) was introduced and evaluated on even more infants (377 high-risk infants) by Ihlen et al. [18]. The 1–5 min video recording of 9–15 weeks corrected age infants were used to predict CP. Pixel movements were tracked using a large displacement optical flow and six body parts (arms, legs, head, and torso) were segmented in a non-automatic way, having two assistance manually annotating the videos. A total of 990 features, including the temporal variation, multivariate empirical mode decomposition (MEMD), and Hilbert–Huang transformation of the six body parts, were extracted for 5 s non-overlapping windows of the videos. Two certified GMA observers rated the videos according to classify FMs (FM−, FM+/−, FM+, FM++) using the GMA as comparison to the model. Forty-one (11%) of 377 infants were diagnosed with CP according to a Decision Tree published by the Surveillance of cerebral palsy in Europe by pediatricians (unaware of the GMA outcome) [96]. CIMA model yielded comparable results to the GMA having 92.7% sensitivity and 81.6% specificity rate in CP prediction. Raghuram et al. [15] introduced a more general analysis by building a predictive model for motor impairment (MI) rather than just a CP prediction. RGB videos of 152 infants (3–5 months) were analyzed to predict MI, defined as Bayley motor composite score <85 or CP. The movement analysis contained a pixel tracking using LDOF, a skin model for segmentation and finally an extraction of movement related features. Using logistic regression and a backward selection method to reduce the feature space, 3 mainly contributing values have been identified. The minimum velocity, mean velocity of the infant’s silhouette, and the mean vertical velocity yielded the best results in MI prediction. The presented automated method performed better (79% sensitivity and 91% negative predictive value (NPV) for MI) than the clinical GMA in relation to MI prediction.

Schmidt et al. [17] relied on 2445 video segments for their study. To reduce the data per input video, they further sampled the segment producing 145 frames per segment video. The authors are the only ones to implement a transfer learning approach, which means that a network trained for another task is reused and adopted. The model was built applying Keras VGG19 [97] and trained on the ImageNet dataset classes. Image features were picked up from Layer eight of VGG19, went through a max-pooling layer and normalized before being presented to an LSTM layer for the classification of the images. They reported 65.1% accuracy using a 10-fold cross validation (CV) for their method. In addition, the model seems to prioritize sensitivity (50.8%) over specificity (27.4%). Summarizing the results, the presented work performs worse compared to previous studies and is not feasible in its preliminary state. Especially, the unbalanced class distribution (approximately 15% natural occurrence rate of CP) makes the training of data intensive neural networks more difficult. Therefore, further investigation is required to check if transfer learning-based approaches are suitable for the problem in hand.

**Depth Cameras:** With the invention of the Microsoft Kinect sensor in 2010, motion tracking has become a relatively easy problem to solve [19,44]. Without much effort, it is possible to compute pose and motion parameters using its 640×480 depth images, which are recorded at 30 frames per second [98]. Olsen et al. [99] introduced a 3D model-based on simple geometries, like spheres and cylinders, to describe the infants body using the Kinect sensor. A stomach body part was matched as the only spatial free object. Remaining parts followed constraints due to a hierarchical model for arms, feet, and the head. While body parameters, like position of the stomach and rotation of the remaining parts, were iteratively improved by the Levenberg Marquardt method [100,101], size parameters for the objects are either given or estimated in the beginning. Using the Kinect sensor, RGB-D videos of 7 infants have been recorded and some frames manually annotated to receive the ground truth of the infants poses. The authors compared a graph-based method and model-based method to estimate the location of the extremities. The performance of the models is estimated by calculating the euclidean distance between the manual annotated points and the estimation of joint locations. It could be shown that the model-based method yielded smoother tracking. Based upon this model, Olsen et al. [19] proposed a method to detect spontaneous movements of infants using motion tracking. They computed several features based on the angular velocities and acceleration from their infants’ model. An RGB-D dataset of 11 infants was analyzed. The labeling consisted of two classes (spontaneous movement or not spontaneous) and was done by one of the authors of this study. They reported good performance of 92–98% accuracy for their sequence segmentation method. Nevertheless, it must be emphasized that the method was evaluated on a very small dataset. Khan et al. [102] proposed a method for monitoring infants at home. They collected RGB data of 10 subjects using an additional RGB camera included in Microsoft Kinect. After data preprocessing, 9 geometric ratio features were computed and then presented to an SVM for classification. A 5-fold cross-validation was performed to validate the system and found to be classified at around 80% accuracy. Although the proposed method shows good results, the number of subjects is critically low, and no healthy infants have been observed as all subjects had movement disorders.

**Pose Reconstruction:** Furthermore, different works have attempted to evaluate the accuracy of Prechtl’s GMA by human experts based upon pose reconstruction models. Therefore, outcomes yielded by the classical GMA based on RGB videos have been compared to experts’ analysis of pose estimations extracted from the same videos. Such reconstructed models anonymize the infant’s person-specific information (for example, faces are disguised) while remaining movement related data to access GMs. Thus, these approaches enable data sharing and reduce privacy concerns in large clinical trials or research projects. Using archived videos from 21 infants (8–17 weeks), a computational pose estimation model was elaborated to extract skeleton information by Marchi et al. [103]. The original and skeleton videos of the 14 low-risk and 7 atypical movement babies were assessed by two blind scorers (qualitative assessment of GMs). An agreement of Cohen’s K of 0.90 between both lead to the conclusion that the skeleton estimation comprises the clinically relevant movement. In comparison, Schroeder et al. [104] recently evaluated a Skinned Multi-Infant Linear Model (SMIL) including 3D body surface additionally to the skeleton of the infant. SMIL model creation consists out of several steps, including background and clothing segmentation, landmark (body, face and hand) estimation and a personalization step, where an initial base template is transferred to the “infant specific shape space by performing PCA on all personalized shapes” [105]. The base template represents an infant-based model instead of just downsampling already existing adult models. A total of 29 high-risk infants (2–4-month corrected age) were recorded for 3 min using Microsoft Kinect V1. A GMA expert rated both (first all SMIL, afterwards all RGB videos) in a randomized order. To evaluate the agreement between general and fidgety movement ratings of the sequences, the Intraclass Correlation Coefficient (ICC) was computed. ICC was 0.874 and 0.926 respectively for GM-complexity and FM. In additions, the authors published the Moving INfants In RGB-D (MINI-RGBD) dataset [106], consisting of SMIL applied to 12 sequences of moving infants up to the age of 7 months. These results suggest that the golden standard for the GMA, which is represented by RGB videos, is similar and thus can be replaced by SMIL. While such abstractions of videos seem to retrain the relevant information and thus look promising, the presented methods did not include a fully automated solution based on AI. Classification rates of machine learning algorithm or DL methods need to be tested on the presented pose estimation models in the future.

**Deep Learning:** With the increasing computing power of Graphics processing units (GPUs) in recent years, the training of Neural Networks (NNs) became possible. These DL approaches aim to learn complex problems in an end-to-end manner using great number of data samples with according class labels (supervised). Although it has been shown that NN can perform excellent results in various tasks, they lack the ability to justify their yielded outcomes. Thus, they are also referred to as black box. DL approaches have been used for GMA based on visual sensors in two different ways. First, NNs can function as pose estimation or other feature extraction method. Secondly, some paper implemented NNs as classifier to directly return the classification output.

**Deep Learning for Pose Estimation:** Chambers et al. [36] built a Convolutional Neural Network to extract the pose of infants. They were the only ones to publish an unsupervised approach as preprint and showed that they can distinguish unhealthy movement from infants based on an NB classifier exclusively trained on healthy children. Therefore, 420 videos of assuming healthy infants were collected from YouTube from which 95 were selected, checking that there is more than 5 s of video data and quality is sufficient to extract pose estimation. The age of the infants was estimated by two physical therapists and averaged for the two resulting values. In addition, a clinical dataset was created to evaluate the model after training. The recorded videos of 19 infants (6 preterm, 13 full-term) were evaluated according to the Bayley Infant Neurodevelopmental Screener by an experienced pediatric physical therapist into different risk groups. It compromises a test for neurological and expressive functions and cognitive processes. The approach compromises OpenPose [107], a Convolutional Neural Network trained to locate joint positions. The author adapted it for infants using YouTube and 17 out the 19 clinical videos with manual annotated joint locations. Using the pose estimation, 38 features (posture, acceleration, velocity, etc.) were extracted to train the NB and check if the individuals in the clinical dataset are part of the (assumed healthy) YouTube set. In other words, they classified infants as unhealthy when their movement was different from the healthy reference dataset. In addition to finding important movement features, a Kruskal–Wallis test between the infants risk groups and the calculated Naive Bayes score show significant association ($\chi^{2}\left(3\right)=29.92,p<0.0001$). While the study offers a promising unsupervised approach to analyze infants’ movements that overcomes the obstacle of collecting sufficient data of unhealthy children, the study faces some problems. First, the clinical dataset of 19 infants seems in terms of participants too small. Secondly, the use of YouTube data could be considered as not reliable for medical diagnostic, especially with missing background information as age and health status of the children. Finally, the chosen unsupervised approach reveals whether infants differ from the healthy reference group but does not make statements how they differ. McCay et al. [24] applied OpenPose on the 12 sequences of the MINI-RGBD dataset. An independent expert annotated the videos using the GMA into categories normal and abnormal. Two pose-based histogram features to retrieve a dense representation of the posture of the infants were introduced. They calculated the Histogram of Joint Orientation 2D (HOJO2D) and Histogram of Joint Displacement 2D (HOJD2D) to train KNN, LDA and an Ensemble classifier (MATLAB, not specified in detail). Using leave-one-out cross validation, a best accuracy of 91.67% was achieved for the Ensemble classifier. The promising feature choice and the good performance results are only compromised by the used dataset which lacks a large number of infants. In addition, the data are synthetic which can introduce a degree of uncertainty in the ground truth and missing information for the classifier.

**Deep Learning for GMA Classification:** McCay et al. [23] extended their work by enhancing the preprocessing pipeline and evaluating different kinds of NN architectures for classification on their feature extraction approach. The confidence score of the OpenPose software was used to find anomalous joint positions and correct them by interpolating successfully interpreted frames. Afterwards, the feature vector computed by HOJO2D, HOJD2D, and a concatenation of both was fed to an NN and CNN architecture and compared to standard machine learning algorithms (DT, SVM, LDA, KNN, Ensemble). They have shown high performance and robustness of the DL approaches. In addition, the CNN and NN architectures yielded better results compared to the standard machine learning algorithms. Tsuji et al. [20] recorded 21 infants and labeled intervals of 30 s according Prechtl’s assessment by the help of a physical therapist. An Artificial Neural Network with a stochastic structure was trained on the resulting dataset containing 4 classes (WMs: 193; FMs: 279; CS: 31; and PR: 66). The proposed method compromises a conversion to grayscale with background subtraction, resulting in a binary image where 0 is coded as background and 1 as infant. Several movement features in the categories movement magnitude, movement balance, movement rhythm, and movement of the body center are extracted afterwards. Features are fed to a Log-Linearized Gaussian Mixture Network (LLGMN) which estimates the probabilistic distribution of every data point. After the training, classification can be given by the highest posterior probability of the model. In addition, a threshold for the entropy is given to identify ambiguous input as additional class (Type 0). This class is also addressed when there is no movement in the data. A classification accuracy of 90.2% for the task normal vs. abnormal motions was achieved. To date they are the only ones to create a model distinguishing 4 types of GMs (WMs, FMs, CS, and PR) and retrieve an accuracy of 83.1%. The proposed model trained on a dataset with more infants and additional movement types could lead to a promising approach to automate the GMA.

In general, most visual-based works so far rely on marker-less approaches. While initially good results could be yielded, most obstacles arise with the limited datasets used. Research and especially deep learning approach could benefit from publicly available large datasets. Privacy concerns could be overcome by transforming video data to 3D infant’s models, like SMIL. So far, an automated recognition system utilizing smartphones has not been evaluated. Yeh et al. [108] and Spittle et al. [109] have already shown, that smartphone videos recorded by instructed parents are valid for clinical GMA. Such a system could provide more people, especially in rural areas, access to GMA. GMA could be used as screening for every newborn and be a benefit for the health system. Moreover, non-intrusive markers created for the infant’s special needs could be used to boost the performance of visual system.

### 5.3. General Movement Assessment Based on Visual and Motion Sensors

The techniques reviewed hitherto use either visual or motion sensors for the detection of infant’s GMs. We have observed that the GMA is mostly based on videos of infants, rated by trained professionals and, as such, is influenced by their subjective sense because of mood, fatigue, social issues, etc. Therefore, it is challenging to imply it in clinical settings and, this is perhaps due to its subjective nature [110]. However, there is an emerging demand for further objective methods [7,12,29,110]. To run-over the drawbacks of the previous approaches, efforts have been made to create a multimodal system consisting of both visual and motion sensors. Berge et al. [110] presented a software tool named enhanced interactive general movement assessment (ENIGMA) for the GMA knowledge extraction and modeling. They acquired video and motion data from the past 15 recordings having normal and abnormal GMs at the fidgety age. To model the features, trained GMA professional guided the knowledge engineer iteratively and incrementally by providing the feedback. They claim that their proposed system suggests a procedure to build an automated system. Moreover, they also proposed a periodicity feature for the detection of FMs. However, they did not provide any quantifiable evaluation on the performance of their proposed feature.

Multi-modality permits for a consistent assessment of GMs and FMs in order to avoid missing data due to occlusion, noise, gestational age, exhaustion, etc. In this section, we reveal the available multimodal approaches for GMA. We split up these techniques on the grounds of fusion level into the *Decision Fusion* and *Feature Fusion* as shown in Figure 3.

**Decision Fusion:** The decision fusion approaches designed to blend the results of various algorithms (or models) into one single called ensemble decision. Numerous methods are proposed [111] to make a single final decision. Among them, majority voting is the general approach to fuse the results of different modalities. In the majority voting scheme, each model gives one vote (i.e., label), and the majority label in the composition is selected as the final decision. It is worthwhile to mention that the reviewed GMA studies [13,21,23,24] used the decision fusion with motion sensors data or visual sensors data.

**Feature Fusion:** Feature fusion is the tactic of joining various modalities features by integrating them into only a high-dimensional feature vector. The integrated features are then used to train and test a classifier. Literature shows that feature fusion practice has higher performance as compared to the decision fusion process. Nevertheless, it can also raise several issues such as the curse of dimensionality and missing data because of the non-availability of the device at a particular time. Techniques such as Principal Component Analysis (PCA) and autoencoders can be used to solve the high-dimensionality problem. There are several methods to deal with the partially or entirely missing data, such as imputing missing values or choosing an algorithm that supports the missing values.

Redd et al. [112] introduced a novel sensing system using 9-axis IMU (Bosch Sensortec Accelerometer + Gyroscope and a Magnetometer) with a sampling rate of 100 Hz. A custom sensor case with an attached triangular array of spherical retro-reflective markers was built. They have produced results by combining IMUs with a marker-based approach. In addition, the authors tried to keep the sensor weight as small as possible (10.25 g), since masses of 14 g do not interfere with fidgety movements [113]. Sensors should be placed on forehead, sternum, left hand, right hand, left foot, and right foot of the infant. However, the system was tested on only one healthy infant at 12 weeks postterm age for which movement data and trajectories were illustrated. To justify and evaluate the system, a machine learning study on a larger dataset should be performed.

Machireddy et al. [34] proposed a multimodal system using visual and motion sensors that integrates marker-based tracking in video images with the IMU measurements. Multiple sensors are used to indemnify for one’s shortcomings. The markers (or color patches) and IMUs are attached to the infant’s hands, legs, and chest with soft bands and vest. From the marker shape, size, and camera calibration, a value for the 3D position is computed. The IMUs and video camera are synchronized simultaneously, and the signals from all sensors are fused using an Extended Kalman filter. They reported 70% classification accuracy on dataset of 20 infants while using train and test data from different limbs. This technique needs to be further analyzed on a larger dataset.

To summarize, we review here two types of fusion levels, such as *decision fusion* and *feature fusion* for infants GMA using motion sensors and visual sensors. Decision fusion combines the outcomes of various algorithms into one single called ensemble decision. In contrast, feature level fusion can combine different modalities data (features) to make a high dimensional feature vector. Nevertheless, the high dimensional feature vector with missing data can raise some issues such as the curse of dimensionality and missing data that can be managed by using dimension reduction and interpolation techniques.

We have summarized the results of reviewed articles in Table 4, Table 5 and Table 6. Table 4 shows the classification results of studies focusing on the general movements. Table 5 represents the classification results of studies focusing on the fidgety movements, and Table 6 lists the combined results of general movements and fidgety movements. All the studies have been ordered by year in their respective Tables.

## 6. Conclusions

In this paper, we presented a review of recent AI approaches that attempt to automate the assessment of general movements in order to overcome the cumbersomeness of (traditional and) clinical GMA. We discussed the advantages and limitations of each type of approaches in their respective sections. In Section 5.1, we have found that motion sensors like accelerometers, gyroscopes, and magnetometers are affordable and sufficiently miniaturized to be placed on infants’ limbs and to record necessary data for the assessment of general and fidgety movements. However, for the purpose of manual annotation the whole process is recorded by cameras for the experts [25,27]. Then, in Section 5.2, we presented that the video data can be useful to track the movement of the limbs to identify the existence of cramped synchronized movements, and also to discover absence of fidgety movements. The marker-based approaches in Section 5.2.1 produce higher accuracies however, placing several markers results in an extra setup time. For example, Meinecke et al. [7] proposed an analysis system for CP in infants by using 20 reflective markers and 7 infrared cameras to capture 3D motion. They produced results with high accuracy (73%). However, such a system is costly and challenging to set up, especially when placing a large number of markers on infants’ little bodies. The work of Berthouze and Mayston [86] used 3 to 4 markers on the shanks of infants in their surface-marker cluster approach for GMA employing 6 cameras. They have placed a relatively small number of markers but still the system is not easily portable and also have high computational complexity which limits their clinical applicability. The authors in [87] presented a cost-effective and easily portable system by using only one digital camera to capture the infant’s movements by placing reflective markers on the joints. They computed and tested different kinematic features extracted from 2D videos.

In contrast, marker-free approaches provide the freedom of using no markers, so they are inexpensive and easy to setup. We have presented several approaches in Section 5.2.2 that use marker-free pose estimation for GMA [21,89]. Most of the approaches employed hand-crafted features [16,22] to quantify the amount of motion for the classification of fidgety and non-fidgety movements. However, the features were sensitive to lightening conditions, cloths, and skin color [14]. Some approaches have used the trajectory of limbs [21,33] to identify the normal and abnormal movements. The authors in [13] worked on a large dataset and presented results using frequency and time-based features. These approaches used 2D videos which may cause reduction in accuracy for the movements that are not performed in the plane perpendicular to the camera. To overcome this problem, several studies have used depth cameras in their research and produced good results. For example, Olsen et al. [99] extracted joints and pose information from RGB-D data for detecting spontaneous movements of infants. In another paper, Olsen et al. [19] presented a model-based approach for tracking infants in 3D. A Deep Learning-based approach [36] is also used on the dataset built upon YouTube videos. The hybrid approaches have also been experimented by incorporating the features extracted from motion and visual data [32] and implemented the motion segmentation methods.

The RGB and depth data have been popularly used for pose and shape estimations that are the building blocks to track the movements of the infants’ limbs. However, we have learned that all the aforementioned approaches are missing extensive learning-based methods using the state-of-the-art classification algorithms and the multimodal sensor setup. We also presume the need of a large purposely collected dataset for GMA that can be used for the learning-based classification approaches. Nevertheless, the privacy of the patients should not be compromised. The SMIL model can be helpful in this regard because it targets the aspect of privacy and provides a very nice idea to generate 3D models of real infants and perform the analysis. Last but not least, we have observed that some of the reviewed studies used more general terms rather than mentioning the standard terms for GMA, for example, normal vs. abnormal sometimes does not emphasize whether the study had dealt with GMs or FMs. In addition, the outcome categories are not compliant with Prechtl’s GMA and the rarer categories like Ch or AF are often not mentioned in the analyses. Therefore, in our opinion, the use of standard terms is more meaningful and would enhance the clarity of the work.

Considering the above-mentioned points, we propose an end-to-end deep learning-based system with the following features:

- The collection of a large dataset of infants for GMA is necessary to implement learning-based approaches.
- The dataset should be comprised of multiple sensor modalities like visual, depth, motion data so that the strength of each modality can be exploited to produce accurate results.
- The privacy preservation techniques should be exercised to conceal the identity of the probands.
- We can use state-of-the-art methods for extracting features, for example, joints information, in visual and depth data.
- The implementation of multi-task learning approach would be beneficial to track the movement of different limbs simultaneously.
- The precise objective of our system is the classification of the infants’ movements into fidgety and non-fidgety.

We believe this approach will help to develop a screening instrument for CP for pediatricians and general practitioners. Then they could detect those infants at highest risk for cerebral palsy (FM) and refer them to specialized centers and start with the adequate therapy as soon as possible. The system would be also beneficial to the parents of high-risk infants living in remote areas to reassure them with a normal result. From the ethical point of view, all affords should deal with developing an AI system that can be implemented in (newly) industrialized countries and developing countries. To support this scientific task, we also take into consideration the publishing of dataset so that research community can use it for the development of further enhanced tools for GMA.

## Figures and Tables

**Table 1:** The literature search strategy (PubMed).

**Infant**	Infants **OR** Newborns **OR** Babies
**AND**	
**Movements**	General Movements **OR** Fidgety Movements **OR** Spontaneous movements
	**OR** Movement estimation **OR** Movement analysis **OR** Motion analysis
**AND**	
**Detection**	Cerebral palsy **OR** Motor impairment **OR** Neurological disorders
**AND**	
**Using**	Machine learning **OR** Computer-based **OR** Video
	**OR** Images **OR** IMU **OR** Motion sensors

**Table 2:** The list of sensors used for the assessment of general movements (GMs) and fidgety movements (FMs).

	GMA Study	Meinecke et al. [7]	Rahmati et al. [32]	Adde et al. [14]	Raghuram et al. [15]	Stahl et al. [16]	Schmidt et al. [17]	Ihlen et al. [18]	Gao et al. [28]	Machireddy et al. [34]	McCay et al. [23]	Orlandi et al. [13]	Olsen et al. [19]	Singh and Patterson [25]	Dai et al. [21]	Heinze et al. [26]	Gravem et al. [27]	Rahmati et al. [33]	Tsuji et al. [20]	Philippi et al. [29]	Karch et al. [31]	Adde et al. [22]	Fan et al. [30]	McCay et al. [24]
Modalities																								
RGB Camera			X	X	X	X	X	X		X	X	X			X			X	X	X		X		X
Vicon System		X																						
Microsoft Kinect								X			X		X											X
Accelerometer														X		X	X						X	
IMU									X	X														
EMTS			X															X		X	X			

**Table 3:** The list of classification algorithms used for the assessment of GMs and FMs.

	Study	Orlandi et al. [13]	Rahmati et al. [32]	Rahmati et al. [33]	Adde et al. [14]	Raghuram et al. [15]	Stahl et al. [16]	Schmidt et al. [17]	Dai et al. [21]	Meinecke et al. [7]	Machireddy et al. [34]	Olsen et al. [19]	Tsuji et al. [20]	Adde et al. [22]	McCay et al. [24]	Ihlen et al. [18]	McCay et al. [23]	Singh and Patterson [25]	Rahmati et al. [32]	Rahmati et al. [33]	Heinze et al. [26]	Gravem et al. [27]	Gao et al. [28]	Machireddy et al. [34]	Fan et al. [30]
CA																									
NB																		X							X
LDA															X	X	X								
QDA										X															
LR		X			X	X								X											
SVM			X				X		X		X	X					X	X	X			X	X	X	X
KNN												X			X		X						X		
DT												X					X	X			X	X			
RF		X							X													X			X
AB		X							X																X
LB		X																							
XGB									X																
LLGMN													X												
CNN								X									X								
PLSR				X												X				X					
DPD																							X		
		Indirect Sensing (via Visual Sensors)																Direct Sensing (via Motion Sensors)							

**Table 4:** Classification results of general movements (GMs) studies.

Ref. & Year	Dataset Information	Features	Method	Results
Meinecke et al. [7]: 2006	**Subjects:** 22 infants (15 healthy,	53 quantitative	**Classification:**	QDA:
	7 high-risk)	parameters, optimal	healthy vs. at-risk	73% acc
	**Age Range**: 44 weeks gestational age	8 selected using	**Validation:**	100% sen
	**Sensor**: Vicon system	cluster analysis	cross validation	70% spe
	**Data**: 92 measurements			
Singh and Patterson [25]: 2010	**Subjects:** 10 premature born babies with	statistical features,	**Classification:**	SVM: 90.46% acc
	brain lesions	temporal features	CS vs. not-CS	NB: 70.43% acc
	**Age Range**: 30–43 weeks gestational age		**Validation:** 10-fold	DT: 99.46% acc
	**Sensor**: Accelerometers		cross validation	
	**Data**: 684,000 samples			
Gravem et al. [27]: 2012	**Subjects:** 10 premature born babies	statistical features,	**Classification:**	SVM/DT/RF:
	**Age Range**: 30–43 weeks gestational age	temporal features	CS vs. not-CS	70–90% avg acc
	**Sensor**: Accelerometers	Total: 166 (features)	**Validation:** 10-fold	90.2% avg sen
	**Data**: Approx. 700,000 samples		cross validation	99.6% avg spe
Fan et al. [30]: 2012	**Subjects:** 10 premature born babies	basic motion features,	**Classification:**	ROC:
	**Age Range**: 30–43 weeks gestational age	temporal features	CS vs. not-CS	72% sen
	**Sensor**: Accelerometers	Total: 84 (features)	**Validation:** 10-fold	57% spe
	**Data**: 98 CS GM segments and 100		cross validation	
	non-CS GM segments			
McCay et al. [24]: 2019	**Subjects:** 12	Histogram-based	**Classification:**	LDA: 69.4% acc
	**Age Range**: up to 7 months	Pose Features,	normal vs. abnormal	KNN(K = 1): 62.50% acc
	**Data**: Synthetic MINI-RGBD dataset of	HOJO2D,	**Validation:** Leave-one	KNN(K = 3): 56.94% acc
	12 sequences	HOJD2D	out cross validation	Ensemble: 83.33% acc
McCay et al. [23]: 2020	**Subjects:** 12	Pose-based fused	**Classification:**	LDA: 83.33% acc
	**Age Range**: up to 7 months	features (HOJO2D +	normal vs. abnormal	KNN(K = 1): 70.83% acc
	**Data**: Synthetic MINI-RGBD dataset of	HOJD2D)	**Validation:** Leave-one	KNN(K = 3): 66.67% acc
	12 sequences		out cross validation	Ensemble: 65.28% acc
				SVM: 66.67% acc
				DT: 62.50% acc
				CNN(1-D): 87.05% acc
				CNN(2-D): 79.86% acc

acc: Accuracy; sen: Sensitivity; spe: Specificity; avg: Average; CS: Cramped Synchronized Movements. ***Note***: we use the classification and output terms as specified in the papers.

**Table 5:** Classification results of fidgety movements (FMs) studies.

Ref. & Year	Dataset Information	Features	Method	Results
Adde et al. [22]: 2009	**Subjects:** 82 infants (n=32 high) and	Motion features, i.e.,	Logistic regression	Triage threshold
	(n = 50 low) risk infants	Quality of motion (Q),	analysis to explore	analysis of the centroid
	**Age Range**: 10–18 weeks	Q_mean_, Q_max_, Q_SD_,	fidgety vs. non-fidgety	of motion C_SD_:
	**Sensor**: Video camera	V_SD_, C_SD_, A_SD_, etc.		90% sen
	**Data**: 137 recordings			80% spe
Adde et al. [14]: 2010	**Subjects:** 30 High-risk infants	Motion features, i.e.,	Logistic regression	ROC Analysis:
	(23–42 weeks)	Quality of motion (Q),	analysis to explore	85% sen
	**Age Range**: 10–15 weeks post-term	Q_mean_, Q_median_, Q_SD_,	motion image features	88% spe
	**Sensor**: Video camera	V_SD_, A_SD_, CPP	for CP prediction	
Stahl et al. [16]: 2012	**Subjects:** 82 infants	Wavelet analysis	**Classification:**	SVM:
	**Age Range**: 10–18 weeks post-term	features from	impaired vs. unimpaired	93.7% acc
	**Sensor**: Video camera	motion trajectories	**Validation:** 10-fold	85.3% sen
	**Data**: 136 recordings		cross validation	95.5% spe
Karch et al. [31]: 2012	**Subjects:** 65 infants (54 neurological	Stereotype score	**Classification:**	ROC:
	disorder, 21 control group)	feature based	CP vs. no-CP	90% sen
	**Age Range**: 3 months	on dynamic time	**Validation:** N/A	96% spe
	**Sensor**: Video Camera, Motion sensors	wrapping		
Philippi et al. [29]: 2014	**Subjects:** 67 infants (49 high-risk,	Stereotype score	**Classification:**	ROC:
	18 low-risk)	of arm movement	CP vs. no-CP	90% sen
	**Age Range**: 3 months post term		**Validation:** NDI	95% spe
	**Sensor**: Video Camera, Motion sensors		including CP vs.	
			no-NDI	
Rahmati et al. [32]: 2014	**Subjects:** 78 infants	Motion features, i.e.,	**Classification:**	Motion segmentation
	**Age Range**: 10–18 weeks post-term	periodicity, correlation	healthy vs. affected	SVM: 87% acc
	**Sensor**: Video camera,	b/w trajectories using	**Validation:**	Sensor data:
	Motion sensors	motion segmentation	cross validation	SVM: 85% acc
Rahmati et al. [33]: 2016	**Subjects:** 78 infants	Frequency based	**Classification:**	Video-based data:
	**Age Range**: 10–18 weeks post-term	features of motion	healthy vs. affected	91% acc
	**Sensor**: Video camera,	trajectories	**Validation:**	Sensor data: 87% acc
	Motion sensors		cross validation	
Machireddy et al. [34]: 2017	**Subjects:** 20 infants	Video camera and	**Classification:**	SVM: 70% acc
	**Age Range**: 2–4 months post-term	IMU signal fusion	FM+ vs. FM−	
	**Sensor**: IMU’s, Video	using EKF	**Validation:** 10-fold	
	camera		cross validation	
Orlandi et al. [13]: 2018	**Subjects:** 82 preterm infants	643 numerical features	**Classification:**	RF: 92.13% acc
	**Age Range**: 3–5 months corrected age	from literature	CP vs. not-CP	LB: 85.04% acc
	**Sensor**: Video camera	regarding GMA	**Validation:** Leave-one	AB: 85.83% acc
	**Data**: 127 Retrospective recordings		out cross validation	LR: 88.19% acc
Dai et al. [21]: 2019	**Subjects:** 120 infants (60 normal &	wavelet & power	**Classification:**	Stacking: SVM/RF/
	60 abnormal behavior)	spectrum, PCA,	normal vs. abnormal	AB → XGBoost
	**Age Range**: 10–12 weeks age	Adaptive weighted	movement	93.3% acc
	**Sensor**: Video camera	fusion	**Validation:** 4-fold	95.0% sen
	**Data**: 120 samples, N/A length		cross validation	91.7% spe
Raghuram et al. [15]: 2019	**Subjects:** Preterm infants	Kinematic features	**Classification:**	LR:
	**Age Range**: 3–5 months post-term		MI vs. no-MI	66% acc
	**Sensor**: Video camera		**Validation:** N/A	95% sen
	**Data**: 152 Retrospective recordings			95% spe
Schmidt et al. [17]: 2019	**Subjects:** infants at risk	Transfer learning, to	**Classification:**	DNN:
	**Age Range**: <6 months	pre-process the video	7 classes,	65.1% acc
	**Sensor**: N/A	frames to detect	**Validation:** 10-fold	50.8% sen
	**Data**: 500 Retrospective recordings	relevant features	cross validation	
Ihlen et al. [18]: 2020	**Subjects:** 377 High-risk infants	990 features describing	**Classification:**	CIMA model:
	**Age Range**: 9–15 weeks corrected age	movement frequency,	CP vs. no-CP	87% acc
	**Sensor**: Video camera	amplitude and	**Validation:** Double	92.7% sen
	**Data**: 1898 (5 s) periods with CP,	co-variation for 5 s	cross-validation	81.6% spe
	18321 (5 s) periods without CP	non-overlapping time		
		periods		

acc: Accuracy; sen: Sensitivity; spe: Specificity; NDI: Neurodevelopment impairment; EKF: Extended Kalman filter; MI: Motor impairment; CP: Cerebral palsy; CIMA: Computer-based infant movement assessment; PCA: Principal Component Analysis; CPP: Cerebral palsy predictor. ***Note***: we use the classification and output terms as specified in the papers.

**Table 6:** Classification results of general movement (GMs) and fidgety movement (FMs) studies.

Ref. & Year	Dataset Information	Features	Method	Results
Heinze et al. [26]: 2010	**Subjects**: 19 healthy, 4 unhealthy	Extracted 32 features	**Classification:**	DT: avg. ODR:
	**Age Range**: Avg. gestational age	as described in [7]	healthy vs. pathologic	89.66% acc
	healthy (39.6) weeks,		**Validation:** Train	avg. PPV 65%
	unhealthy (29.25) weeks		test split	avg. NPV 100%
	**Sensor**: Accelerometers			
1st m.	**Subjects:** 9 healthy, 4 unhealthy	Extracted 32 features	**Classification:**	Classification results:
	**Age Range**: mean age (SD) in days	as described in [7]	healthy vs. pathologic	ODR: 89%, PPV: 75%
	healthy 24 (±4), unhealthy 29 (±16)			NPV: 100%
2nd m.	**Subjects:** 17 healthy, 4 unhealthy	Extracted 32 features	**Classification:**	Classification results:
	**Age Range**: mean age (SD) in days	as described in [7]	healthy vs. pathologic	ODR: 88%, PPV: 50%
	healthy 87 (±20),unhealthy 77 (±28)			NPV: 100%
3rd m.	**Subjects:** 15 healthy, 4 unhealthy	Extracted 32 features	**Classification:**	Classification results:
	**Age Range**: mean age (SD) in days	as described in [7]	healthy vs. pathologic	ODR: 92%, PPV: 71%
	healthy 147 (±14),unhealthy 143 (±11)			NPV: 100%
Olsen et al. [19]: 2015	**Subjects:** 11 infants	Angular velocities	**Classification:**	SVM/DT/KNN:
	**Age Range**: 1–6 months	and acceleration	SP vs. not-SP	92–98% acc
	**Sensor**: Microsoft Kinect,	of the joints	**Validation:**	
	**Data**: 50,000 labelled frames		cross validation	
Gao et al. [28]: 2019	**Subjects:** 34 infants (21 typical	Temporal features,	**Classification:**	KNN: 22% avg acc
	developing (TD), and 13 with	PCA for dimension	TD vs. AM	SVM: 79% avg acc
	perinatal stroke)	reduction	**Validation:** 10-fold	DPD: 80% avg acc
	**Age Range**: 1–6 months post-term		cross validation	No-DPD: 70% avg acc
	**Sensor**: IMU’s			
Tsuji et al. [20]: 2020	**Subjects:** 21 infants (3 full-term, 16 low	Motion features from	**Classification:**	LLGMN:
	birth weight, 2 unknown status)	video images using	normal vs. abnormal	90.2% acc
	**Age Range**: N/A	background difference	movements	
	**Sensor**: Video camera	and frame difference	**Validation:**	
	**Data**: 21 video recordings		cross validation	

acc: Accuracy; sen: Sensitivity; spe: Specificity; avg: Average; SP: Spontaneous; TD: Typical development; AM: Abnormal movements; PCA: Principal Component Analysis; LLGMN: Log-Linearized Gaussian Mixture Network, 1st m: Measurement around the first month; 2nd m: Measurement around the third month; 3rd m: Measurement around the fifth month; ODR: Overall detection rate; PPV: Positive predictive value; NPV: Negative predictive value; ***Note***: we use the classification and output terms as specified in the papers.

## Abbreviations

The following abbreviations are used in this manuscript:

AB	AdaBoost
AI	Artificial Intelligence
ANN	Artificial Neural Networks
BINS	Bayley Infant Neurodevelopmental Screener
CIMA	Computer-based Infant Movement Assessment
CNN	Convolutional Neural Network
Ch	Chaotic General Movements
CP	Cerebral palsy
CS	Cramped Synchronized General Movements
CV	Cross Validation
DL	Deep Learning
DPD	Discriminative Pattern Discovery
DT	Decision Tree
DWT	Discrete Wavelet Transform
EKF	Extended Kalman Filter
EMTS	Electromagnetic Tracking System
FM	Fidgety Movement
GMA	General Movement Assessment
GM	General Movement
GPU	Graphics Processing Units
HAR	Human Activity Recognition
IMU	Inertial Measurement Unit
KCF	Kernel Correlation Filter
KNN	K-Nearest Neighbor
LDA	Linear Discriminant Analysis
LDOF	Large Displacement Optical Flow
LEDs	Light Emitting Diodes
LLGMN	Log-Linearized Gaussian Mixture Network
LOO-CV	Leave-One-Out Cross-Validation
LR	Logistic Regression
LSR	Least Square Regression
MEMD	Multivariate Empirical Mode Decomposition
MI	Motor Impairment
NB	Naive Bayes
NNs	Neural Networks
NPV	Negative Predictive Value
PCA	Principal Component Analysis
PLSR	Partial Least Square Regression
PR	Poor Repertoire General Movements
QDA	Quadratic Discriminant Analysis
RF	Random Forests
RMDS	Root Mean Square Deviation
SMIL	Skinned Multi-Infant Linear Model
SVM	Support Vector Machine
